# Stress cardiac magnetic resonance in an outpatient setting: a four year experience in > 1000 patients

**DOI:** 10.1186/1532-429X-13-S1-P81

**Published:** 2011-02-02

**Authors:** Golmehr Ashrafpoor, Susanna Prat- Gonzalez, Amir-Ali Fassa, Yannick Yannick Magliano, Alain Naïmi, Juan Sztajzel

**Affiliations:** 1 Cardiology Service, Geneva University Hospitals, Geneva, Switzerland; 2 Centre de Diagnostic Radiologique de Carouge, Geneva, Switzerland

## Introduction

Only few data are available on the use of stress cardiac magnetic resonance (CMR) in a totally outpatient setting.

## Purpose

In this sense, we present our four year experience in 1017 stress CMR examinations performed in our outpatient institution.

## Methods

We reviewed the data of all patients who were referred for stress CMR (1.5 Tesla) from February 2006 to February 2010 to the Centre de Diagnostic Radiologique de Carouge, an outpatient imaging center. Standard protocol consisted of: 1) assessment of myocardial function at rest; 2) pharmacological stress induced either by dobutamine (protocol of 10, 20, 30, 40 µg/kg/min during 3 minutes with atropine if necessary) until achieving submaximal heart rate ([220-age] x 0.85), or by adenosine (protocol of 140 µg/kg/minute during 3 minutes followed by a bolus of 10 ml of gadolinium at 4 ml/second "first pass"); 3) assessment of myocardial scar and/or viability by delayed enhancement (DE) sequences.

## Results

During the study period 1017 patients were referred for stress CMR. The test could be performed in 994 patients (98 %), 635 males (64 %), mean age 63±12 years [range 17-91 years]). Stress was induced using adenosine in 650 patients (65 %) and with dobutamine in 345 patients (35%). Mean duration with adenosine was 46 ± 8 minutes and with dobutamine 58 ± 8 minutes. The test could not be carried out in 23 patients (2%), in 17 during the first year and in 6 patients between years 2, 3 and 4. Claustrophobia was the cause in 18 patients and excessive thoracic diameter in 5 patients. No ischemia or infarction was found in 656 patients (66%), while isolated ischemia was found in 42 patients (4%) and ischemia in the presence of an infarction in 83 patients (8%). Infarction without ischemia was found in 203 patients (20%). Complications occurred in 21 patients (2%) including: supraventricular tachycardia or unsustained ventricular tachycardia (8 patients), chest pain (4 patients), suspected allergic reaction to gadolinium (1 patient), dizziness (1 patient), vomiting during dobutamine infusion (1 patient) and hypotension (1 patient). No other complications occurred.

## Conclusions

In our large outpatient population, stress CMR could be safely performed and it was well tolerated. The feasibility of the examination directly correlated with the learning curve of our team. One third of all patients had an abnormal test.

